# Rhamnogalacturonan, a chemically-defined polysaccharide, improves intestinal barrier function in DSS-induced colitis in mice and human Caco-2 cells

**DOI:** 10.1038/s41598-018-30526-2

**Published:** 2018-08-16

**Authors:** Daniele Maria-Ferreira, Adamara Machado Nascimento, Thales Ricardo Cipriani, Arquimedes Paixão Santana-Filho, Paulo da Silva Watanabe, Debora de Mello Gonçales Sant´Ana, Fernando Bittencourt Luciano, Karla Carolina Paiva Bocate, René M. van den Wijngaard, Maria Fernanda de Paula Werner, Cristiane Hatsuko Baggio

**Affiliations:** 1 Department of Pharmacology, Universidade Federal do Paraná, Curitiba, Brazil; 2 Department of Biochemistry and Molecular Biology, Universidade Federal do Paraná, Curitiba, Brazil; 3 Department of Biosciences and Physiopathology, Universidade Estadual de Maringá, Maringá, Brazil; 4 Department of Animal Science, School of Life Sciences, Pontifícia Universidade Católica do Paraná, Curitiba, Brazil; 5 Tytgat Institute for Liver and Intestinal Research, Department of Gastroenterology and Hepatology, Academic Medical Center, Amsterdam, The Netherlands

## Abstract

Natural polysaccharides have emerged as an important class of bioactive compounds due their beneficial biological effects. Here we investigated the protective and healing effects of rhamnogalacturonan (RGal) isolated from *Acmella oleracea* (L.) R.K. Jansen leaves in an experimental model of intestinal inflammation in mice and in heterogeneous human epithelial colorectal adenocarcinoma cells (Caco-2). The findings demonstrated that RGal treatment for 7 days reduced the severity of DSS-induced colitis by protecting mice from weight loss, macroscopic damage and reduction of colon length. When compared to the DSS group, RGal also protected the colon epithelium and promoted the maintenance of mucosal enterocytes and mucus secreting goblet cells, in addition to conserving collagen homeostasis and increasing cell proliferation. In an *in vitro* barrier function assay, RGal reduced the cellular permeability after exposure to IL-1β, while decreasing IL-8 secretion and claudin-1 expression and preserving the distribution of occludin. Furthermore, we also observed that RGal accelerated the wound healing in Caco-2 epithelial cell line. In conclusion, RGal ameliorates intestinal barrier function *in vivo* and *in vitro* and may represent an attractive and promising molecule for the therapeutic management of ulcerative colitis.

## Introduction

Ulcerative colitis (UC) is a chronic relapsing and idiopathic disease characterized by a diffuse inflammation that affects the colonic mucosa with bloody diarrhea as a predominant symptom^1^. Epidemiological studies suggest that the occurrence of this disease is increasing worldwide, and the reason remains unknown^2^. Currently, it has been pointed out that UC presents multifactorial character, resulting from the interaction between genetic predisposition, environmental factors, gut microbiota and immune response^3^. Nevertheless, despite the relevance and the impact of the disease on health and social life of patients, the etiology of UC remains unclear and the current treatment options, which includes corticosteroids, aminosalicylates, immunomodulators and monoclonal antibodies, often lacks clinical effectiveness and has manifold detrimental side effects. Thus, the development of new treatment approaches and management strategies to treat patients according to the severity and extent of UC requires an urgent investigation^4^.

Taking into account, recent studies have shown that medicinal plant-derived extracts, herbs and dietary components such as flavonoids have anti-colitis activity, through controlling the levels of inflammatory mediators associated with the severity of active UC^5–7^. Furthermore, polysaccharides purified from plants and fungi have emerged as an important class of natural products due to the diversity of their biological activities in several models of diseases^8–10^. Accordingly, it has been shown that prebiotic multifiber mixture and non-digestible polysaccharides are able to reduce the inflammation and symptoms associated with IBD^1^.

For instance, our research group demonstrated the gastroprotective properties of rhamnogalacturonan (RGal), a chemically-defined polysaccharide easily obtained in high yield from leaves of *Acmella oleracea* (L.) R.K. Jansen^11^, that is commonly used as ingredient for food in northern Brazil. Interestingly, our previous studies revealed that this polysaccharide protected the gastric mucosa against acute lesions induced by ethanol and promoted gastric ulcer healing in a chronic ulcer model induced by acetic acid in rats. These effects were due to the increase of cellular proliferation and gastric mucus content, associated with the reduction of inflammatory parameters and oxidative stress, without toxicological effects after sub chronic exposure^12^.

Considering this, the purpose of our study was to investigate the protective and healing effects of RGal in an acute experimental model of intestinal inflammation chemically induced by dextran sulfate sodium (DSS) in mice. Furthermore, to substantiate possible effects on barrier function, we employed *in vitro* assays using heterogeneous human epithelial colorectal adenocarcinoma (Caco-2) cells.

## Results

### RGal improves disease activity and colonic damage induced by DSS

It is well known that the DSS is directly toxic to colonic epithelium leading to severe illness, characterized by shortening of the colon, bloody diarrhea and sustained weight loss, the main signal manifestations of UC^13^. To evaluate the potential effect of RGal in DSS-induced colitis, the animals were treated with daily oral doses of RGal (3, 10 and 30 mg/kg) or vehicle (water, 1 mL/kg), once a day, from day 1 to day 7.

**Figure 1:**
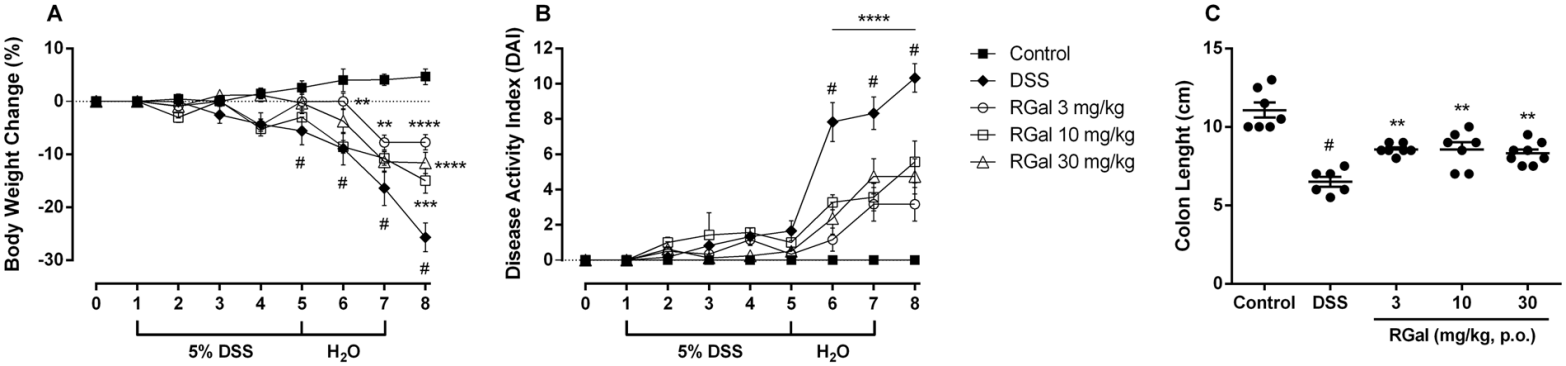
RGal protects mice against DSS-induced colitis. Effect of RGal treatment on (**A**) body weight change, (**B**) disease activity index, and (**C**) colon length. Mice were orally treated with vehicle (Control or DSS groups: water, 0.1 mL/kg) or RGal (3, 10 or 30 mg/kg) for 7 days, once a day. Results are expressed as mean ± S.E.M. (n = 8) and analyzed using ANOVA followed by Bonferroni’s test. ^#^*P* < 0.05 compared to Control group, *P < 0.05, **P < 0.01, ***P < 0.001 and ****P < 0.0001 compared to DSS group.

When compared to healthy mice (naïve control group), animals that received DSS in drinking water and were treated with vehicle started to lose weight at day 5 (6.54%) and this extended up to the day 8 (27.30%), (Fig. 1A). The same holds for the DAI (Disease Activity Index), where the DSS group presented visible blood in the stool, as well as a colon length reduction (61%) when compared to the naïve control group (Fig. 1C,D). Moreover, the animals also presented occult blood in the feces (Supplementary Table S1).

The anti-colitis effect promoted by RGal does not assume a dose-response relationship, both 3, 10 and 30 mg/kg achieved similar effects. Taking into account, we decided to work with a safer dose (10 mg/kg) in the subsequent experiments, considering the aggressiveness of the ulcerative colitis model induced by DSS. In the mice group submitted to DSS-induced colitis, RGal treatment (10 mg/kg) significantly reduced the body weight loss (day 8, 51%) and DAI from day 5 (28%) to day 8 (46%) when compared to the DSS group (Fig. 1A,B). RGal also prevented the reduction of colon length (RGal: 9.1 ± 0.2 cm) when compared to DSS group (DSS: 7.1 ± 0.2 cm) (Fig. 1C,D) and diminished the presence of occult blood in the feces when compared to DSS group (Supplementary Table S1).

### RGal treatment decreases the cellular infiltration and improves microscopic damage in colonic tissue

**Figure 2:**
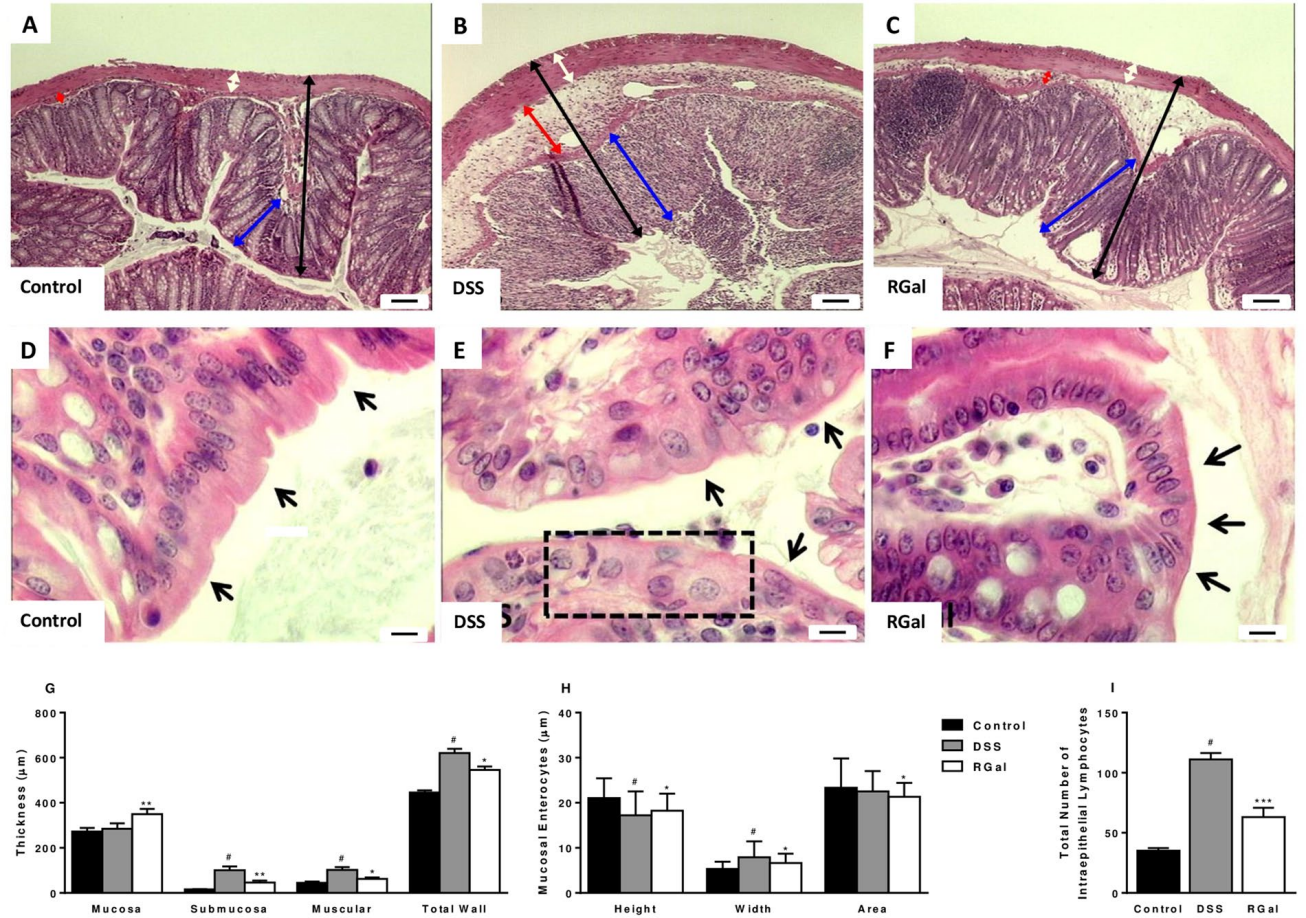
RGal improves histological parameters. Hematoxylin and eosin-stained sections of colons. (**A**–**C**) Histopathological changes in mucosa (blue arrow), submucosa (red arrow), muscular layer (white arrow) and total colonic wall (black arrow), ×40 (bars = 100 µm). (**D**–**F**) Evaluation of height, width and area of mucosal enterocytes (arrows). Disarrangement of enterocytes is indicated by the dashed box. (**G**,**H**) Measurement of colonic layers thickness and of mucosal enterocytes of colons. (**I**) Quantification of intraepithelial lymphocytes. Mice were orally treated with vehicle (Control or DSS groups: water, 0.1 mL/kg) or RGal (10 mg/kg) for 7 days, once a day. Results are expressed as mean ± S.E.M or mean ± S.D. (n = 4–8) and analyzed using ANOVA followed by Bonferroni’s test. ^#^*P* < 0.05 compared to Control group, **P < 0.01 and ***P < 0.001 compared to DSS group.

Previous studies have shown that the colonic damage induced by DSS directly relate to cellular infiltration into the intestinal mucosa^14^. For this reason, we next evaluated if the protective effect of RGal in DSS-mediated colitis was associated with alterations in inflammatory cell numbers in colonic tissue. For this, colons were processed for histological analysis and quantification of intraepithelial lymphocytes. The DSS sections of H&E stain revealed a destruction of colonic tissue with histopathological changes in the mucosal, submucosal, muscular layer and colonic wall. However, the histological evaluation of colons from RGal-treated mice revealed a significant increase in mucosal thickness (22%) and decrease in the thickness of the submucosal, muscular layer and total colonic wall (54, 38 and 18%, respectively) when compared to the DSS group (Fig. 2A,G). This result was accompanied by the maintenance of mucosal enterocytes, width and area (Fig. 2B,H). Also, the data showed that the number of intraepithelial lymphocytes increased significantly in the DSS group (217%) when compared to the control group (35.0 ± 2.2 total number) and the RGal treatment reduced intraepithelial lymphocytes infiltration an average of 43%, when compared to the DSS group (111.0 ± 5.4 total number) (Fig. 2I).

**Figure 3:**
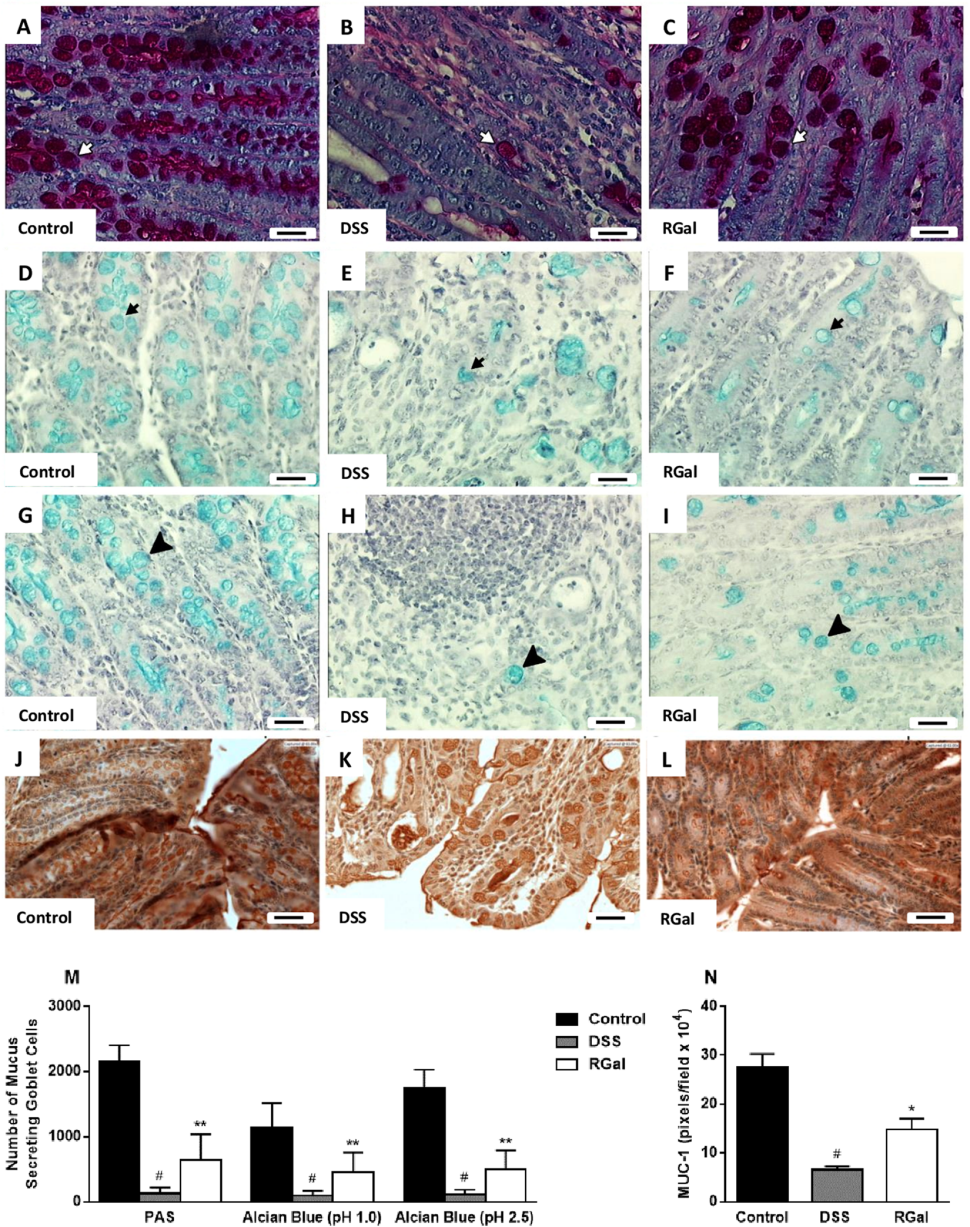
RGal preserves the colonic goblet cells and the expression of MUC-1. Histochemical staining of colons for (**A**–**C**) neutral mucin-like glycoproteins (PAS, white arrows), (**D**–**F**) and (**G**–**I**) acid mucin [Alcian blue: pH 2.5 (black arrow) and pH 1.0 (black arrowhead)], ×400, bars = 50 μm. (**J**–**L**) Immunohistochemical staining for MUC-1 in colons, ×400, bars = 20 μm. (**M**,**N**) Quantification of mucus secreting goblet cells and MUC-1 expression, respectively. Mice were orally treated with vehicle (Control or DSS groups: water, 0.1 mL/kg) or RGal (10 mg/kg) for 7 days, once a day. Results are expressed as mean ± S.E.M or mean ± S.D. (n = 4–8) and analyzed using ANOVA followed by Bonferroni’s test. ^#^*P* < 0.05 compared to Control group, *P < 0.05 and **P < 0.01 compared to DSS group.

We also observed the maintenance of the total number of mucus secreting goblet cells with PAS and AB staining methods (Fig. 3A–I and M).

### RGal preserves the mucus layer and promotes cell proliferation

**Figure 4:**
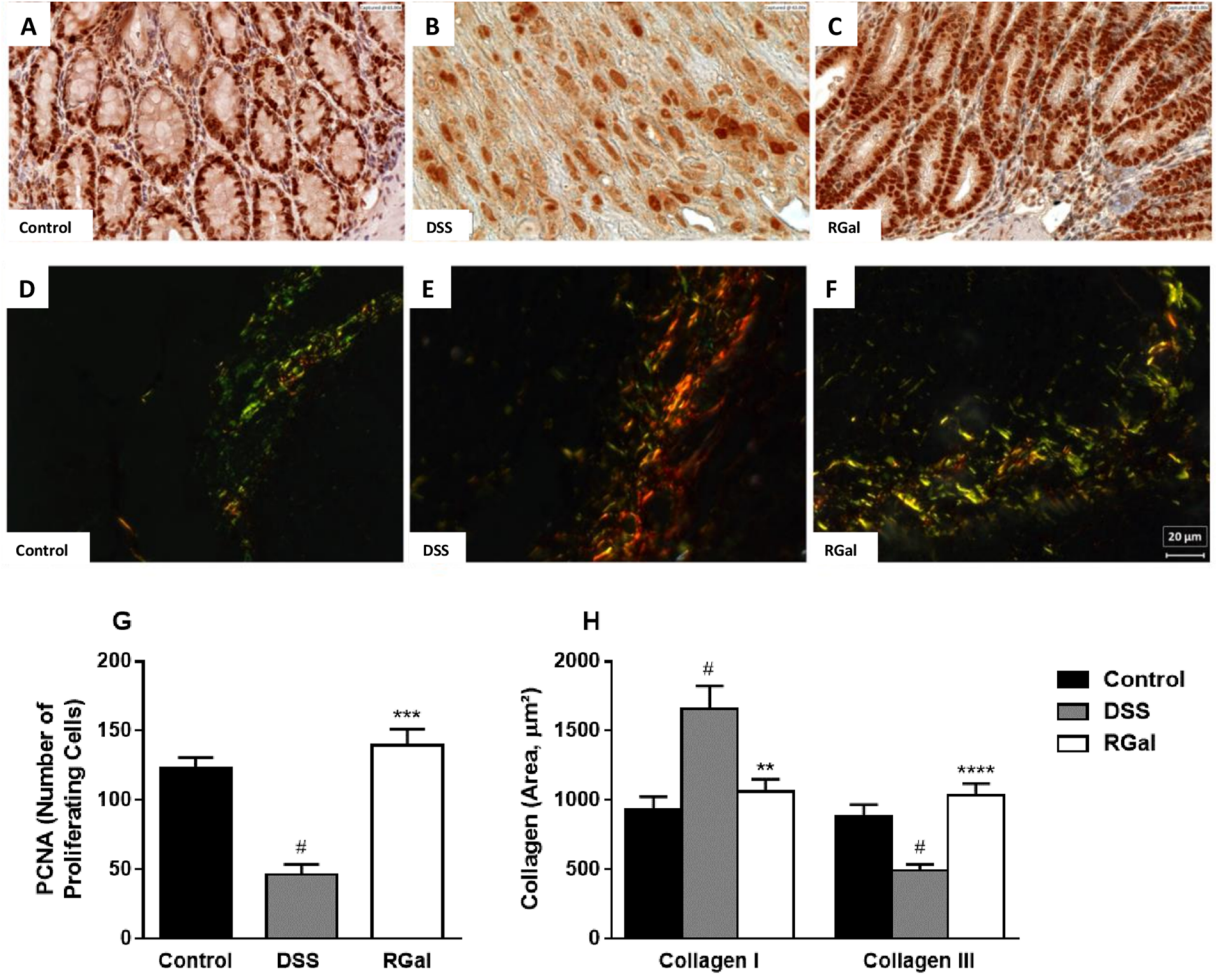
RGal promotes cell proliferation and attenuates colonic fibrosis. (**A**–**C**) Immunohistochemical staining for PCNA and (**D**–**F**) *Sirius Red* staining for collagen I and III in colons, ×400, bars = 20 μm. (**G**,**H**) Quantification of proliferating cells (PCNA) and collagen, respectively. Mice were orally treated with vehicle (Control or DSS groups: water, 0.1 mL/kg) or RGal (10 mg/kg) for 7 days, once a day. Results are expressed as mean ± S.E.M (n = 4) and analyzed using ANOVA followed by Bonferroni’s test. ^#^*P* < 0.05 compared to Control group, **P < 0.01, ***P < 0.001 and ****P < 0.0001 compared to DSS group.

To confirm previous data that showed an increase in the amount mucus goblet cells into the colonic tissue of RGal-treated animals, we also performed immunohistochemical analysis to determine the expression profile of MUC-1, a cell surface mucin involved in cellular signaling, adhesion, growth and immunological modulation^15^. According to the immunohistochemical patterns, the results in Fig. 3K,N shows that the administration of DSS promoted a decrease in mucin-1 levels (71%) when compared to the control group (Fig. 3J,N). However, oral administration RGal (10 mg/kg) increased the labeling for MUC-1 by 127% (Fig. 3L,N) when compared to the DSS group (DSS: 6.51 ± 0.79 pixels/field x 10^4^). In addition, colon of DSS animals showed a significant decrease in the immunoreactivity for PCNA (63%) (Fig. 4B,G), which is characterized by brown color and indicates proliferating cells, when compared to control group (C: 122.70 ± 8.09 number of cells per field of view) (Fig. 4A,G). However, RGal treatment was able to promote an average of 302% increase in PCNA immunoreactivity when compared to the control group (Fig. 4C,G).j

### Treatment with RGal attenuates intestinal fibrosis

Mice treated with DSS develop colitis that is characterized by transmural inflammation and fibrosis, which represents a serious complication of inflammatory bowel diseases (IBD). For this reason, we quantified the collagen deposition in the colonic tissue. The study of colonic sections stained with Sirius red demonstrated an extensive type III collagen deposits (70.28%) and reduction in type I collagen (55.88%) in DSS mice (Fig. 4D,E and H). On the other hand, the treatment with RGal normalized the proportions of collagens when compared to the DSS group (RGal collagen III: 1062.00 ± 87.52 μm, collagen I: 1036.00 ± 84.68 μm) (Fig. 4F,H).

### RGal stimulates wound healing in Caco-2 cells

**Figure 5:**
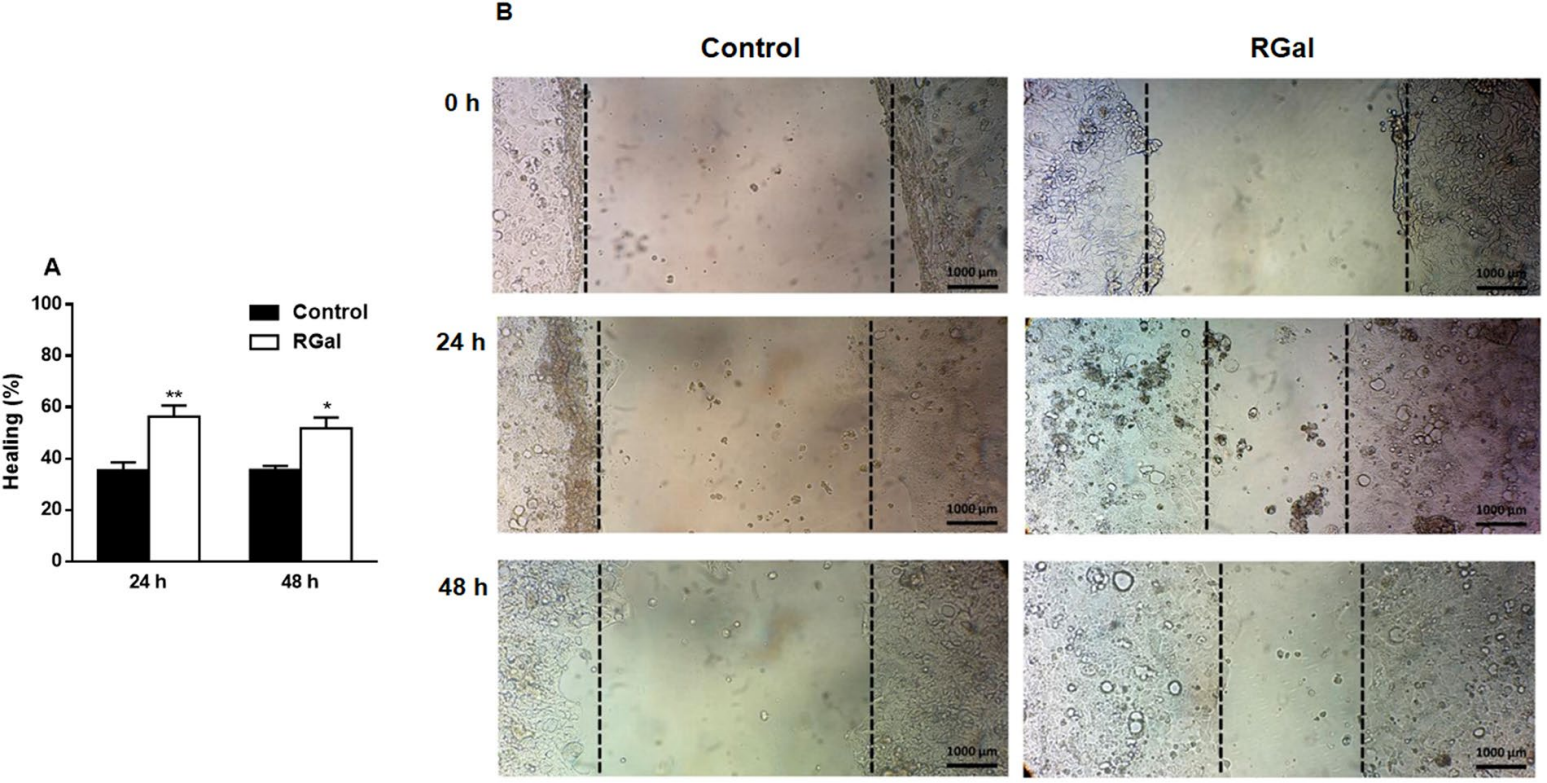
RGal accelerates the wound closure in Caco-2 cells. (**A**) Percentage of wound healing at 24 and 48 h after scratch (*n* = 3, in triplicate). (**B**) Representative images of scratched areas at 0, 24 and 48 h, ×10, bars = 1000 μm. Confluent cell monolayers were wounded with a pipette tip and incubated with medium alone (DMEM, control) or RGal (1000 μg/mL) for 48 h. Wound healing was photographed at 0, 24 and 48 h after scratch. Results are expressed as mean ± S.E.M. (n = 3) and analyzed using ANOVA followed by Bonferroni’s test. *P < 0.05 and **P < 0.01 compared to Control group.

Once we observed an improvement of the colonic tissue in the *in vivo* model of DSS-induced colitis we also determined whether the RGal is capable of accelerating Caco-2 cells healing. RGal (1000 µg/mL) greatly accelerated wound closure of scratched Caco-2 cell monolayers 24 and 48 h after wounding (Fig. 5A,B). The polysaccharide promoted lesion closure by 84 and 45% at 24 and 48 h, respectively, when compared to the control group (DMEM 24 h: 25.45 ± 2.82% and DMEM 48 h: 55.61 ± 4.00%).

### RGal attenuates intestinal epithelial barrier dysfunction induced by IL-1β

**Figure 6:**
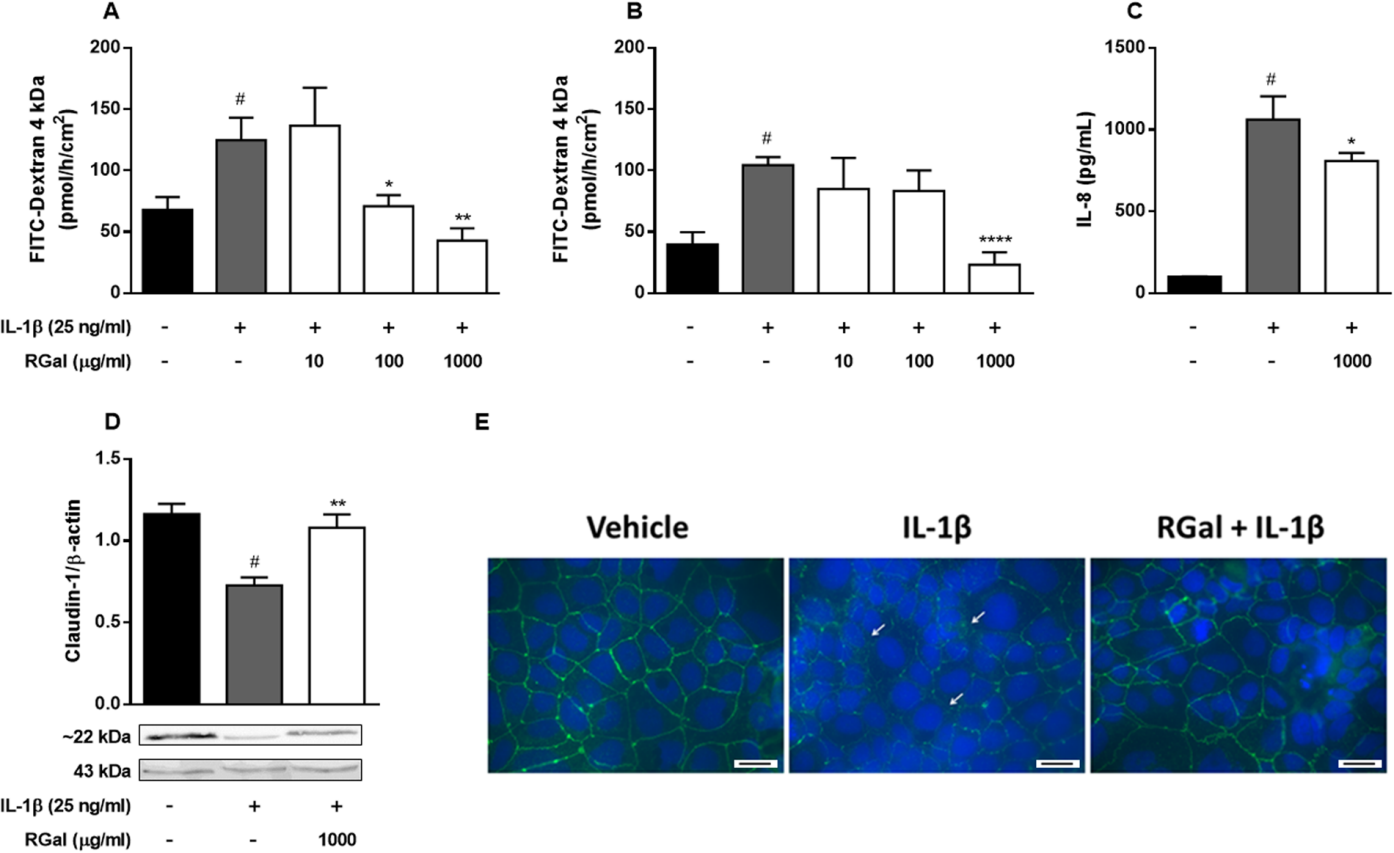
RGal attenuates the intestinal epithelial barrier dysfunction induced by IL-1β *in vitro*. (**A**) Permeability assay, IL-1β added to the apical side and (**B**) permeability assay, IL-1β added to the basolateral side. (**C**) Quantification of IL-8 secretion by ELISA and (**D**) Claudin-1 expression by western blot assay. The blot images have been cropped for conciseness. The full-size blots are presented in Supplementary Fig. S1. (**E**) Immunofluorescence staining for occludin (white arrows shows the occludin disruption) and DAPI (nuclei), ×400, bars = 100 μm. Cells were treated with vehicle or RGal (10, 100 and 1000 µg/mL) and after 6 h, IL-1β (25 ng/mL) was added for 72 h. Results are expressed as mean ± S.E.M. (n = 8) and analyzed using ANOVA followed by Bonferroni’s test. ^#^*P* < 0.05 compared to Vehicle group. *P < 0.05, **P < 0.01 and ****P < 0.0001 compared to IL-1β group.

The treatment of Caco-2 cell monolayer with IL-1β leads to disruption of intestinal barrier function *in vitro* and causes loss of barrier integrity^16^. To further determine if the RGal promotes a protection of intestinal epithelial barrier, we assessed the intestinal epithelial barrier dysfunction in IL-1β stimulated Caco-2 cells. When Caco-2 cells were stimulated with IL-1β in the inner well, the FD4 passage increased 83% when compared to the vehicle group (67.8 ± 10.5 pmol/h/cm^2^). However, RGal (100 and 1000 µg/mL reduced the permeability by 43 and 65%, respectively (Fig. 6A). Additionally, when cells were stimulated with IL-1β in the outer well, the cellular permeability increased 162% when compared to the vehicle group (39.8 ± 9.5 pmol/h/cm^2^), and RGal pretreatment (1000 µg/mL) reduced the cellular permeability in 77% when compared to IL-1β group (Fig. 6B).

### RGal treatment inhibits IL-1β-induced secretion of IL-8

After being exposed to IL-1β, the Caco-2 cells express and release many inflammatory mediators in the cell medium, such as IL-8. Under our experimental conditions, stimulation of Caco-2 cells with IL-1β (25 ng/mL) for 72 h increased the secretion of IL-8 in 949% when compared to the vehicle group (101.1 ± 0.8 pg/mL). On the other hand, RGal (1000 µg/mL) reduced in 24% the levels of IL-8, when compared to the IL-1β group (Fig. 6C).

### RGal prevents morphological disrupton of tight junction (TJ) proteins induced by IL-1β

Increased secretion of proinflammatory cytokines might cause paracellular permeability, due primarily to the disruption of tight junction proteins such as claudins and occludin^16^. To verify the integrity of intercellular junctional complexes (claudin-1, and occludin) we performed western blot and immunofluorescence assay. As expected, the vehicle group expressed high amounts of claudin-1 protein. After IL-1β exposure, the expression decreased 36% and the pre-stimulation of cells with RGal was able to prevent the reduction of expression of claudin-1 in 42% (Fig. 6D and Supplementary Fig. S1) when compared to IL-1β group.

Following 72 h exposure to IL-1β, fluorescent staining showed that intercellular junctions of cells markedly lost the well-defined bright green occludin outline, which suggested the disruption of the tight junction protein occludin. On the other hand, pre-stimulation of cells with RGal prevented this loss, showing uniform and continuous outlines (Fig. 6E).

### Cytotoxicity effects

Importantly, our results revealed that neither RGal nor IL-1β were cytotoxic for Caco-2 cells in all experimental sets (Supplementary Fig. S2).

### Bioaccessibility of RGal

**Figure 7:**
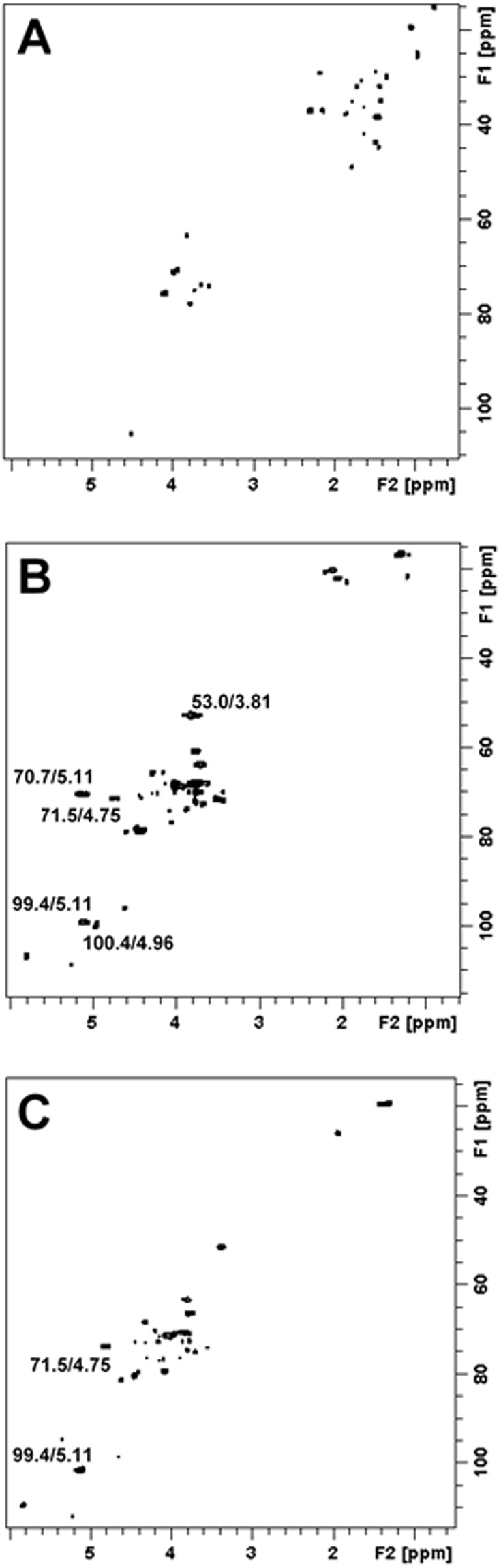
Simulated digestion does not cleave the main structure of RGal. Partial 2D ^1^H-^13^C HSQC NMR spectra of (**A**) duodenal simulated media, (**B**) RGal and (**C**) RGal plus duodenal simulated media. The experiments were acquired according to material and methods section, and chemical shifts referenced to TSP (δ = 0.00 ppm).

The bioaccessible fraction is the amount of an ingested compound that is available for absorption in the body after digestion. In this context, we investigated the effect of simulated human digestive fluids on the chemical structure of RGal. ^13^C/^1^H HSQC NMR experiments showed that treatment with such human digestive fluids caused de-esterification of the methyl-esterified galacturonic acid units (6-O-Me-GalA) of RGal, according to disappearance of the correlations at 100.4/4.96, 70.7/5.11 and 53.0/3.81, of C1/H1, C5/H5 and –CO_2_-CH_3_ of 6-O-Me-GalA units, respectively. The ^13^C/^1^H correlations at 99.4/5.11 and 71.5/4.75 are from C1/H1 and C5/H5 of non-esterified galacturonic units (Fig. 7B,C). The pH changes caused by treatment with the simulated digestive fluids probably caused de-esterification of RGal. Despite this, the others ^13^C/^1^H correlations did not change after the treatment, indicating that RGal still remained as a polysaccharide.

## Discussion

**Figure 8:**
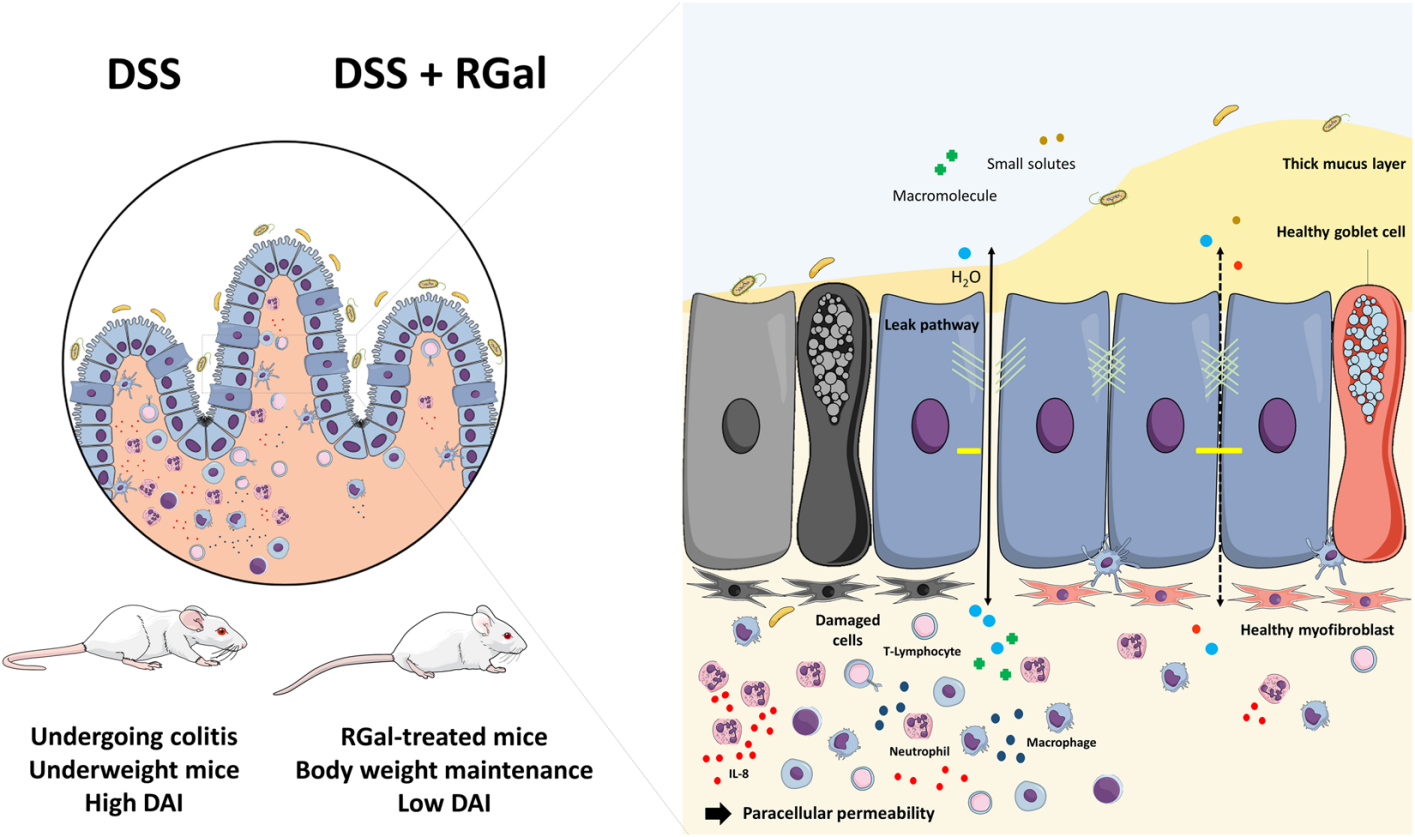
Schematic summary of the results.

The development of new effective agents with minimal or without adverse effects is a constant search in the treatment of UC, that is a multifactorial disorder. Here, we describe a new role for RGal in decreasing the intestinal inflammatory process chemically induced by DSS in mice. We also demonstrated the reduction of epithelial permeability and cytokine secretion as well as the maintenance of the tight junction integrity in Caco-2 cells. The schematic summary of our results is shown in Fig. 8.

The DSS model is characterized by a general inflammatory process directly associated with body weight loss, bloody diarrhea and histopathologic changes that mimic some clinical aspects of UC in humans^17^. Indeed, we observed that DSS induced body weight loss and caused a significant increase of DAI score, which was accompanied by colonic shortness and bloody stool. Interestingly, RGal treatment markedly improved these features in mice with DSS-induced colitis showing that this polysaccharide might constitute a potential alternative to prevent or delay the progression of inflammatory conditions in the gut. Currently, the conventional therapy for UC, such as glucocorticoids, is used in the active phase and for maintaining remission. However, the adverse effects remain a major concern for prolonged usage, which also may include peptic ulceration and wound healing impairment^18^. In this sense, treatment with RGal not only improved the general parameters of the disease in DSS model but we demonstrated previously that RGal also protected the gastric mucosa against formation of acute lesions and accelerated the healing of chronic ulcers in rats^12^, suggesting that RGal could be better option to the treatment of UC.

One of the most typical events underlying the pathogenesis of UC is the influx of inflammatory cells to the intestinal mucosa. The anti-inflammatory effects presented by RGal could be evidenced by the significantly reduction of intraepithelial lymphocytes infiltration. Additionally, we had previously demonstrated that RGal reduced the MPO and TNF-α levels in an experimental model of chronic ulcer induced by acetic acid^12^, reinforcing that RGal displays an important role in the control of inflammatory cell influx and consequently promotes an amelioration of colonic inflammation in DSS-induced colitis model. It has been demonstrated that it is directly involved to colonic damage, which in turn is associated with mice malnutrition and consequently body weight loss^19^. Including, microscopic examination of the colon sections revealed a marked intestinal epithelial destruction by DSS, with histopathological changes in the mucosa, submucosa, muscular layer and colonic wall, which was reversed in RGal-treated mice. It is noteworthy that RGal treatment was also able to preserve the mucosal enterocytes, which are essential to transport and process soluble antigens in the gut^20^, as well as mucus secreting goblet cells, confirmed by the immunohistochemical labeling for MUC-1, ensuring the normal mucus secretion and consequently the homeostasis between the host and the microbiota^21^. Although MUC-2 is the most abundant intestinal mucin produced by goblet cells, previous studies have shown that the mucus layer is significantly decreased in animals that have the inactivated MUC-1 gene, also indicating the importance of cell surface MUC-1^22^.

Another important phenomenon involved in the pathogenesis of IBD due to the inflammatory process in the mucosa is the fibrosis. Complications of fibrosis can lead to the loss of tissue function and may contribute to the formation of intestinal stenosis^23^. In our model of DSS-induced colitis, we observed an increase of collagen III and decrease of collagen I due to the inflammatory process in the colon of mice. On the other hand, RGal therapy promoted the maintenance of homeostasis among collagens showing that perhaps, as a consequence of the anti-inflammatory action, RGal could be also able to prevent intestinal fibrosis and thus avoid the development of stenosis and obstruction of the colon.

In addition to the aforementioned characteristics, apoptosis has also been reported in IBD. It has already been proposed that the imbalance between apoptosis and proliferation of new cells causes relevant damage to the epithelial barrier. This hypothesis is supported by the finding that both increased apoptosis and decreased proliferation of new epithelial cells occurs in the acute phase of DSS-induced colitis^24^. In view of this, during cell proliferation, which represents a critical phase to form new crypts, PCNA plays a key role in the cell renewal cycle and can work as a tissue marker^25^. Here, we observed that RGal was able to increase the expression of proliferating cells, being evidenced by the increase in brown staining relative to animals treated with the vehicle alone. Therefore, as we already have been demonstrated in another model of gastrointestinal disease, the restoration of gastric mucosal cells continuity promoted by RGal occurs through epithelial cell proliferation^12^.

To reinforce our preclinical data and confirm the protective effects of RGal, we initiated complementary *in vitro* experiments using Caco-2 cells. The intestinal epithelium is constantly exposed to an enormous diversity of stimuli, and the lesion of the intestinal epithelial cells is constant and almost unavoidable. In patients with UC, intestinal lesion is typical and during this process, the mucosal cells migrate to cover the injured area, regardless of the proliferation, trying to maintain the integrity of the intestinal barrier^7^. Using a classic *in vitro* cicatrization model conducted with Caco-2 cells, we demonstrated that RGal treatment facilitated the cicatrization and/or closure process of cell monolayers and it may be associated to the proliferation or migration of epithelial intestinal cells, giving support to the *in vivo* data and reinforcing the potential application of RGal in the tissue healing process.

The repeated intestinal epithelial injury that occurs in the mucosa of UC patients leads to development increased paracellular permeability of intestinal epithelial cells and, consequently, epithelial barrier dysfunction. Accordingly, *in vitro* experiments conducted with human Caco-2 cells stimulated with IL-1β provides a good experimental model to evaluate it^26^. Il-1β is a pro-inflammatory cytokine that is notable elevated in intestinal mucosa under inflammatory conditions. In addition, this cytokine causes an increase in intestinal TJ permeability, contributing to the development of intestinal inflammation^27^. In intestinal Caco-2 cells, for instance, Il-1β decreases transepithelial electrical resistance (TER)^28^, which is in part mediated by increased expression of MLCK and MLC phosphorylation and decreased expression and redistribution of the tight junction occludin. Additionally, the administration of a zonulin peptide inhibitor (AT-1001), prevented the development of intestinal inflammation in IL-10^−/−^ mice, suggesting that defective small intestinal permeability may be an important event in the development of UC^29^.

Accordingly, our measurements of FITC-dextran permeation clearly show that RGal markedly reduced the Caco-2 permeability after IL-1β stimulation, maintaining the cellular monolayer integrity. Also, we observed that RGal preserved the expression of claudin-1 and occludin, suggesting that the polysaccharide can prevent the abnormal altered permeability and consequently infections, and the establishment of inflammation in the gut. Additionally, the levels of pro-inflammatory chemokines, such as IL-8, have been found in the intestinal tissue of UC patients and, together with other cytokines, are responsible in initiating, mediating and perpetuating intestinal inflammation^30^. Here, we demonstrated that RGal elicited a significant inhibitory effect on the IL-1β-induced IL-8 release, which may be reflected on the decreased number of infiltrating cells observed in *in vivo* models.

Although the dataset presented demonstrates the important biological effect of RGal related to the colonic protection and regeneration, the underlying mechanisms remain unclear. Interestingly, despite the pH changes of digestive fluids have caused de-esterification of RGal, the original main structure remains the same, showing that probably the polysaccharide is not digestible and may be available for fermentation in the large intestine. We can assume that RGal beneficial properties are product of indirect and direct ways of action. Indirectly, RGal acting as a substrate for bacterial fermentation in the large intestine and leading to the production of short-chain fatty acids (SCFAs) including acetate, propionate and butyrate. These SCFAs have been demonstrated in regulating intestinal immunity through the inhibition of histone deacetylases and the activation of G-protein coupled receptors such as GPR41, GPR43 and GPR109A^31^. In addition, it has been demonstrated that butyrate can regulates prostaglandin production, thus stimulating epithelial mucin 2 (MUC-2) and membrane-linked (MUC-1, MUC-3, MUC-4) mucins expression^32,33^, which could explain why we observed more mucin (layer thickening and less breakdown by increased mucin stability) in our results. Another interesting finding is that activation of MUC1 is also associated with gene programming for cell proliferation and survival^34^, a characteristic observed in animals treated with RGal.

On the other hand, RGal was also able to promote healing, reduced intestinal permeability, decreased secretion of IL-8, as well as maintaining the integrity of epithelial junction proteins in experiments *in vitro* in a simple culture system without the presence of bacteria. Despite these direct effects on epithelial cells are less explored, it is evident that RGal may be eliciting its effect through activation of a receptor, such as Toll-like receptors (TLRs). Indeed, some studies showed that the TLR signaling is involved in non-prebiotic effects of oligosaccharides^35,36^. It was demonstrated that TLR2 stimulation activates protein kinase Cα (PKCα) and δ (PKCδ), enhancing the TER and the translocation of ZO-1^37^. In addition, Wu *et al*.^38^ found that the activation of PKCδ was linked to the improvement of intestinal epithelial barrier function by dietary prebiotics (non-digestible polysaccharides). Altogether, the literature and our results point out to a combine way of action of polysaccharides, highlighting RGal, in which the involvement of different signaling pathways converging to the restoration of the colonic tissue integrity. However, further experiments need to address whether those underlying mechanisms are involved in RGal effects or corroborate to the beneficial effects of RGal. With this in mind, RGal may represent a promising molecule for drug development to the treatment of ulcerative colitis.

## Material and Methods

### Rhamnogalacturonan isolation and characterization

Similar to our previous publication on the structure of rhamnogalacturonan (RGal), the polysaccharide was isolated from leaves of *A. oleracea* (L.) R.K. Jansen^11^. Briefly, leaves of *A. oleracea* were extracted with water and the aqueous extract was treated with excess EtOH providing a crude precipitate of polysaccharides. Then, the precipitate was submitted to freezing-thawing until no more precipitate appeared. The soluble portion was treated with acetic acid, which resulted in a soluble and an insoluble fraction. The soluble fraction was composed of uronic acid, galactose, arabinose, rhamnose and glucose in a 15:2:1:1:0.5 molar ratio (molecular weight = 226 000 g/mol).

### Animals

Experiments were performed using adult female Swiss mice (20–30 g), maintained in a controlled-temperature and luminosity environment (22 ±  2 °C, 12 h light/dark cycle). Mice were housed 10 per cage with wood shaving bedding with free access to pelleted food (Nuvilab CR-1, Quimtia S/A, Brazil) and tap water, and they were acclimated to the laboratory environmental conditions one week before the beginning of the experiments. After the experiments, the animals were euthanized by overdose of lidocaine [(4 mg/kg, intraperitoneal (i.p.)] and thiopental (100 mg/kg, i.p.). All protocols were performed upon approval by the Committee of Animal Experimentation of the Universidade Federal do Paraná (CEUA/BIO–UFPR, approval number 863) and were strictly performed in accordance with the “Guide for the Care and Use of Laboratory Animals” (National Research Council, 2011)^39^.

### Induction and assessment of DSS colitis

**Figure 9:**
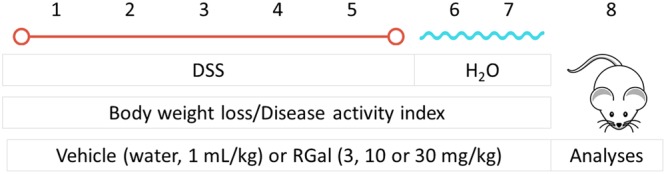
Representative image of experimental protocol used for the induction of ulcerative colitis in mice.

The experimental protocol used for the induction of ulcerative colitis was conducted as previously described^19^ with minor modifications and briefly shown in Fig. 9. The animals were challenged with 5% DSS (Dextran Sulphate Sodium, Molecular weight: 40000 Da, TdB Consultancy) solution in drinking water for 5 consecutive days, which was replenished daily. At the end of fifth day, animals received only normal drinking water for 2 days and at the eighth day after starting the treatments, they were euthanized, and their colons were excised. Control group received only drinking water for 7 days.

### Pharmacological treatments and Disease Activity Index (DAI)

Animals were divided into the following treatments groups: (i) naïve control group, that received only drinking water, (ii) DSS group treated with vehicle [C: water, 1 mL/kg, *per oral* (p.o.)] and (iii) DSS group treated with RGal (10 mg/kg, p.o.). Individual mice were monitored daily to determine the Disease Activity Index (DAI), according to changes of weight loss, stool consistency and occult blood^40^.

The scores were described as follows: weight loss was graded as 0 if body weight increased or remained within 1% of the baseline; 1 for a 1–5% loss; 2 for a 5–10% loss; 3 for a 10–15% loss; or 4 for weight loss >15%. The stool consistency was graded as 0 for no diarrhea; 2 for loose stool that did not stick to the anus; and 4 for liquid stool that did stick to the anus. The presence of fecal blood received a value of 0 when assigned for none, 2 for moderate, and 4 for gross bleeding. At the end of experiment, the colons were collected, washed with saline (0.9%), the lengths were measured and then the tissues were immediately stored at −80 °C for further analysis.

### Histological analysis

For analysis of microscopic damage, the distal portion of each colon was excised and immediately fixed in Alfac solution (85% alcohol 80 °GL, 10% of formaldehyde at 40% and 5% glacial acetic acid) for 16 h and then transferred to 70% ethanol, embedded in paraffin wax and then sectioned at 7 µm thickness before being deparaffinized. Slides were stained using hematoxylin and eosin stain (H&E) to analyze the histopathological changes and to quantify the intraepithelial lymphocytes^41^. Periodic Acid Schif (PAS) and Alcian Blue standard techniques were used to analyze the mucus and goblet cells. *Sirius Red* staining was used for evaluation of type I and III collagen.

### Immunohistochemistry analysis

Immunohistochemistry for mucin 1 (MUC-1) and proliferating cell nuclear antigen (PCNA) was performed on 7 µm thickness paraffin-embedded sections from the colons of mice. Sections were blocked with 1% BSA for 30 min at room temperature and incubated overnight at 4 °C with primary antibodies anti-MUC-1 (rabbit polyclonal IgG, 1:100, Santa Cruz Biotechnology) and anti-PCNA (rabbit polyclonal IgG, 1:100, Santa Cruz Biotechnology). Subsequently, the sections were washed with 1% BSA/PBS and incubated with peroxidase conjugated secondary antibody (goat anti-rabbit, 1:100 Santa Cruz Biotechnology) at room temperature in a humid chamber for 1 h. Peroxidase binding sites were detected by staining using chromogen diaminobenzidine (DAB Substrate Kit, BD Pharmingen™). In all steps the sections were washed three times with PBS. At the last step, the slides were dehydrated and counterstained with Mayer’s hematoxylin. After, the sections were observed and photographed with a slide scanner from MetaSystems (MetaViewer^®^ version. 2.0.100).

### Cell culture

Human epithelial colorectal adenocarcinoma (Caco-2) cells line, purchase from ATCC (Manassas, VA), were grown to 100% confluence in 75 cm^2^ plastic flasks using Dulbecco´s modified Eagle´s medium (DMEM) supplemented with 10% fetal bovine serum (FBS), 2 mM L-glutamine, 1% penicillin and streptomycin and non-essential amino acids (Gibco™). The cells were kept at 37 °C in a 5% CO_2_ environment. Caco-2 cells were subculture after partial digestion with 0.25% trypsin and 0.9 mM EDTA in Ca^2+^ and Mg^2+^ free PBS every 5–7 days.

### *In vitro* scratch assay

To evaluate the intestinal mucosal healing, we used a classic *in vitro* wound healing assay, which consists in creating a “scratch” in a cell monolayer. Caco-2 cells were grown to confluence (3 days) on 6-well culture plates at a density of 2 × 10^5^ cells/well. A linear scratch was made in each well using a 200 µL sterile pipette plastic tip, perpendicular to a black line drawn on the underside of the plate for reference, creating a cell-free area. Posteriorly, to remove detached cells and debris, wounded monolayers were washed with PBS and incubated in 3 mL of FBS-free culture medium containing RGal at 1000 µg/mL at 37 °C. Images of each scratch were captured at 0, 24 and 48 h with a digital camera on an inverted microscope (Olympus) at 10X magnification. The wound closure analysis was made from edge to edge using the ImageJ software. For all treatments, the wound at 0 h was assigned as 100% and the percentage of wound healing at 24 and 48 h was compared to each cell treatment at 0 h^42^.

### Assessment of epithelial barrier integrity

To conduct the epithelial barrier integrity experiments, cells were seeded into polycarbonate filter membranes (0.4 μm pore size, 0.6 cm^2^ filter area, Corning^®^), in 12-transwell permeable support at a density of 2 × 10^4^ cells/well and grown for 21 days for differentiation before the experiments. Cells were preincubated with vehicle (sterile water) or RGal (10, 100 and 1000 µg/mL), and 6 h later stimulated with IL-1β (25 ng/mL). In separate experiments, IL-1β was added to the apical and basolateral compartments. Following 72 h incubation, the medium was removed, the cells were washed twice with PBS and the apical medium was replaced with fluorescein isothiocyanate-dextran [FITC-Dextran 4 kDa (FD4), Sigma]. Samples were collected from the basal compartment at 0, 30 and 60 min and the fluorescence (a mean of 10 measurements per sample) was determined in opaque black 96-well Nunc^TM^ plates at an excitation and emission wavelengths of 490 and 520 nm, respectively, using a FLX-800 fluorimeter (Bio-Tek Instruments Inc., Winooski, VT, USA). Fluorescence of cell culture medium samples were extrapolate from the standard curve using serial dilutions of FITC-Dextran (0–1000 nM) and the results were expressed as pmol/h/cm^2^^ 43^.

### Determination of IL-8 levels

Samples were collected from the basal compartment 72 h after IL-1β incubation and were centrifuged at 16000 × g for 10 min. The levels of IL-8 were evaluated using enzyme-linked immunosorbent assay (ELISA) kit according to the manufacturer’s recommendations (R&D Systems, Minneapolis, MN, USA). Absorbance was measured using a microplate reader (Bio-Tek Instruments Inc., Winooski, VT, USA) at 490 nm. Chemokine levels were extrapolated from the IL-8 standard curve (0–2000 pg/mL) and the results were expressed as pg/mL.

### Western Blotting

Cells were collected and homogenized in RIPA buffer containing Tris (1 M), NaCl (5 M), NP-40, EDTA (0.5 M), DTT (0.1 M), PMSF (0.1 M), and protease inhibitor cocktail (Roche Complete and Roche Phosstop). The homogenate was centrifuged at 3000 × g for 10 min at 4 °C, and the supernatants were stored at −70 °C for further analysis. DTT and bromophenol blue concentrations were adjusted to 100 mM and 0.1% w/v, before running on 12.5% denaturing polyacrylamide gels prior transfer. Membranes were probed sequentially with rabbit anti-human claudin-1 (1:1000, Invitrogen). Additionally, blots were probed with mouse anti-human β-actin (1:1000, Cell Signaling) as a loading control for epithelial proteins and this was used to enable normalization of all protein reactivities to ensure validity of quantitative blotting data. Membranes were incubated with their respective peroxidase conjugated anti-IgG secondary antibody (1:1000, Invitrogen) and then subsequently incubated with the enhanced chemiluminescence system (ECL Plus kit, Amersham GE Healthcare Life Sciences). Blots were carried out under identical conditions for each experiment/antibody, scanned (Bio-Rad^®^) and quantified densitometrically with ImageJ^®^ software and normalized to β-actin for equal epithelial protein loading.

### Immunofluorescence assay

The cells were cultured in slides with 8-well chambers for 21 days and after the treatments the immunofluorescence technique was performed to verify the expression of occludin. The slides were washed with PBS and fixed with 4% paraformaldehyde in PBS for 15–20 min at 4 °C. The cells were then washed again with PBS and incubated with blocking buffer containing 10% fetal bovine serum and 0.05% Tween for 1 h. After, 200 μl of the anti-occludin rabbit polyclonal antibody (1:1000, Invitrogen) was added in each well and incubated for 2 h at room temperature. The wells were washed with PBS and incubated for 1 h in the dark with 200 μl of the goat anti-rabbit IgG secondary antibody conjugated to Alexa Fluor^®^ 488 (1:1000, Thermo Fisher Scientific). Finally, the slides were washed with PBS and prepared with anti-fade assembly medium (DAPI [4′,6-Diamidino-2-Phenylindole, Dihydrochloride], BD Pharmingen) and observed under a fluorescence microscope (Leica DMZ 6000B).

### MTS assay

Cell viability was determined by MTS assay using CellTiter 96^®^ AQueous MTS Reagent Solution (Promega, Madison, WI, USA). The reduction of MTS tetrazolium salt (3-(4,5-dimethylthiazol-2-yl)-5-(3-carboxy-methoxyphenyl)-2-(4-sulfophenyl)-2H-tetrazolium) by metabolically active cells into its reduced formazan form was assessed according to manufacturer’s instruction. Briefly, 21 days after differentiation, cells were incubated with vehicle, RGal (1000 µg/mL) or IL-1β (25 ng/mL) for 72 h. Subsequently, cells were washed with PBS and incubated with fresh medium at 37 °C for 1 h in the presence of MTS substrate before measuring the absorbance at 490 nm with a spectrophotometer (Bio-Tek Instruments Inc., Winooski, VT, USA).

### *In vitro* digestion model

The static *in vitro* digestion model to simulate the human gastrointestinal tract was performed according to a previously reported study with some modifications^44^. Digestive solutions were warmed to 37 ± 1 °C prior to their use. RGal was dissolved in sterile water and added to 50 mL polyethylene tubes to give a final concentration of 100 mg/mL. Digestion started by adding 0.6 mL of artificial saliva [10 mL of KCl (89.6 g/L), 10 mL of KSCN (20 g/L), 10 mL of NaH_2_-PO_4_ (88.8 g/L), 10 mL of Na_2_SO_4_ (57 g/L), 1.7 mL of NaCl (175.3 g/L), 20 mL of NaHCO_3_ (84.7 g/L), 8 mL of urea (25 g/L) and 290 mg of α-amylase, completed to 0.5 L and the pH adjusted to 6.8]. The solution was homogenized in a vortex for 5 s and 10 mL of water was added. Then, pH was immediately adjusted to 2 with 1 N HCl and 0.05 mL of pepsin solution (0.04 g/mL in 0.1 N HCl) was added. Samples were incubated at 37 °C for 2 h in a water bath with rocking agitation (100 rpm) (Dubnoff TE-053, TECNAL, Piracicaba, SP, Brazil). The duodenal compartment was simulated by adding 20 mL of water, adjusting the pH to 6.5 with 1 N NaHCO3 and adding 0.125 mL of a solution containing pancreatin (4 g/L) and bile salts (25 g/L), respectively. The mixture was homogenized and re-incubated at 37 °C for 2 h in the water bath with rocking shaker at 100 rpm. Then, aliquots were lyophilized to evaluate the RGal concentrations in *in vitro* bioaccessibility during gastric and duodenal simulated digestion by NMR analyses.

### NMR spectroscopy

Deuterium oxide (D_2_O, 99.9% D) and 3-trimethylsilyl-^2^H_4_-propionic acid sodium salt (TSP) were purchased from Cambridge Isotope Laboratories, Inc. (Miami, U.S.A.) and from Sigma-Aldrich (St. Louis, MO). Spectra were obtained from solutions in D_2_O (580 µL plus 20 µL of TSP 1 mg/mL at 30 °C, using TSP as reference (δ = 0.00 ppm), using a Bruker AVANCE *III* NMR spectrometer operating at 14.1 Tesla (600.13 MHz for ^1^H) equipped with a QXI probe with gradient on the z-axis (Bruker Biospin, Germany). 2D ^13^C/^1^H multiplicity-edited HSQC NMR experiments were carried out using heteronuclear correlation via double inept transfer with decoupling during acquisition, using trim pulses in inept transfer with multiplicity editing during the selection step (hsqcedetgpsisp2.2). The 2D HSQC experiments were recorded for quadrature detection in the indirect dimension, using 8 scans per series of 2 K × 256 W data points with zero filling in F1 (4 K) prior to Fourier transformation^42^. Data processing and integration were performed using the software Topspin version 3.1 (Bruker Biospin, Rheinstetten, Germany). ^13^C/^1^H correlations were assigned according to Nascimento *et al*.^11^.

### Statistical analysis

All data were expressed as means ± S.E.M. Statistical analysis was performed using Kruskal–Wallis followed by Dunn’s test for non-parametric data, one-way or two-way ANOVA followed by Bonferroni´s test for parametric data. All analysis was conducted using GraphPad Prism 6 software (GraphPad Software Inc., San Diego, CA, USA). Differences with p < 0.05 were considered statistically significant.

## Electronic supplementary material

Dataset 1

## Data Availability

The datasets generated during and/or analyzed during the current study are available from the corresponding author on reasonable request.
