# Supplementary material for: Rhamnogalacturonan, a chemically-defined polysaccharide, improves intestinal barrier function in DSS-induced colitis in mice and human Caco-2 cells
Source: Sci Rep. 2018 Aug 16;8:12261. doi: 10.1038/s41598-018-30526-2 (PMC6095889; doi:10.1038/s41598-018-30526-2)
Supplement: Supplementary file 1 — Dataset 1 [file 41598_2018_30526_MOESM1_ESM.doc]

**Rhamnogalacturonan, a chemically-defined polysaccharide, improves intestinal barrier function in DSS-induced colitis in mice and human Caco-2 cells**

Daniele Maria-Ferreira^1,2^, Adamara Machado Nascimento^2^, Thales Ricardo Cipriani^2^, Arquimedes Paixão Santana-Filho^2^, Paulo da Silva Watanabe^3^, Debora de Mello Gonçales Sant´Ana^3^, Fernando Bittencourt Luciano^4^, Karla Carolina Paiva Bocate^4^, René M. van den Wijngaard^5^, Maria Fernanda de Paula Werner^1*^, Cristiane Hatsuko Baggio^1*^

^1^ Department of Pharmacology and ^2^ Department of Biochemistry and Molecular Biology, Universidade Federal do Paraná; Curitiba, Brazil

^3^ Department of Biosciences and Physiopathology, Universidade Estadual de Maringá; Maringá, Brazil

^4^ Department of Animal Science, School of Life Sciences, Pontifícia Universidade Católica do Paraná, Curitiba, Brazil

^5^ Tytgat Institute for Liver and Intestinal Research, Department of Gastroenterology and Hepatology, Academic Medical Center, Amsterdam, The Netherlands

***Corresponding authors:**

C.H. Baggio, PhD (current address)

Department of Physiology and Pharmacology

Cumming School of Medicine

University of Calgary

3330 Hospital Dr. NW

Calgary, AB, T2N 4N1, Canada

Phone: +1 403 220 4306

E-mail: crisbaggio@gmail.com

M.F.P. Werner, PhD

Department of Pharmacology

Sector of Biological Sciences

Federal University of Parana

PO Box 19031

Curitiba, PR, 81531-980, Brazil.

Phone: +55 41 3361 1721

E-mail: mfernanda.werner@ufpr.br

**Supplementary materials**

**Supplementary Table S1.** RGal treatment decreases the fecal occult blood.

| **Control**  **(water)** | **5% DSS + Vehicle**  **(1 mL/kg, p.o.)** | **5% DSS + RGal**  **(10 mg/kg, p.o.)** |
| --- | --- | --- |
| + | - | + |
| + | + | + |
| + | + | - |
| + | + | + |
| + | + | - |
| + | + | - |

The + and - symbols were used for the appearance of reddish color representing presence or absence of occult blood, respectively.

**Supplementary Figure S1. RGal reduces claudin-1 protein in Caco-2 cells.** Full size blot of all claudin-1 blots. Cells were treated with vehicle or RGal (1000 µg/mL) and after 6 h, IL-1β (25 ng/mL) was added. Seventy-two hours after, lysates were homogenized in RIPA buffer and the membranes were probed with anti-human claudin-1 (1:1000, Invitrogen) and anti-human β-actin (1:1000, Cell Signaling). The treatment of the cells and the molecular weight of claudin-1 and actin are indicated in the blots. The labeling is always the same for cropped and full-size blots.


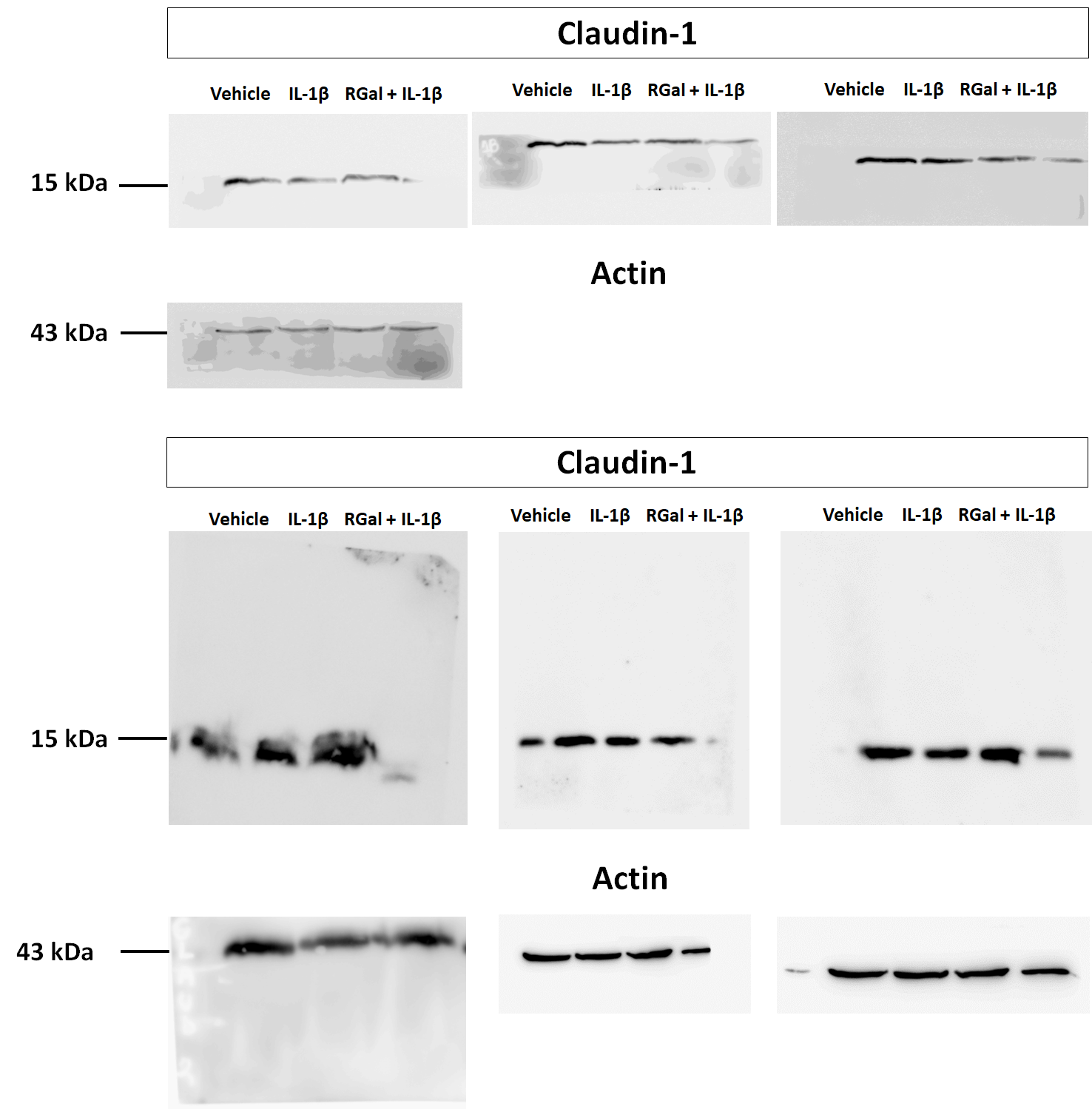


**Supplementary Figure S2. RGal is not toxic to Caco-2 cells.** Cells were treated with vehicle, RGal (10, 100 and 1000 µg/mL) or IL-1β (25 ng/mL). Results are expressed as mean ± S.E.M. (n = 8) and analyzed using ANOVA followed by Bonferroni’s test. ^#^ *P* < 0.05 compared to Vehicle group.

**
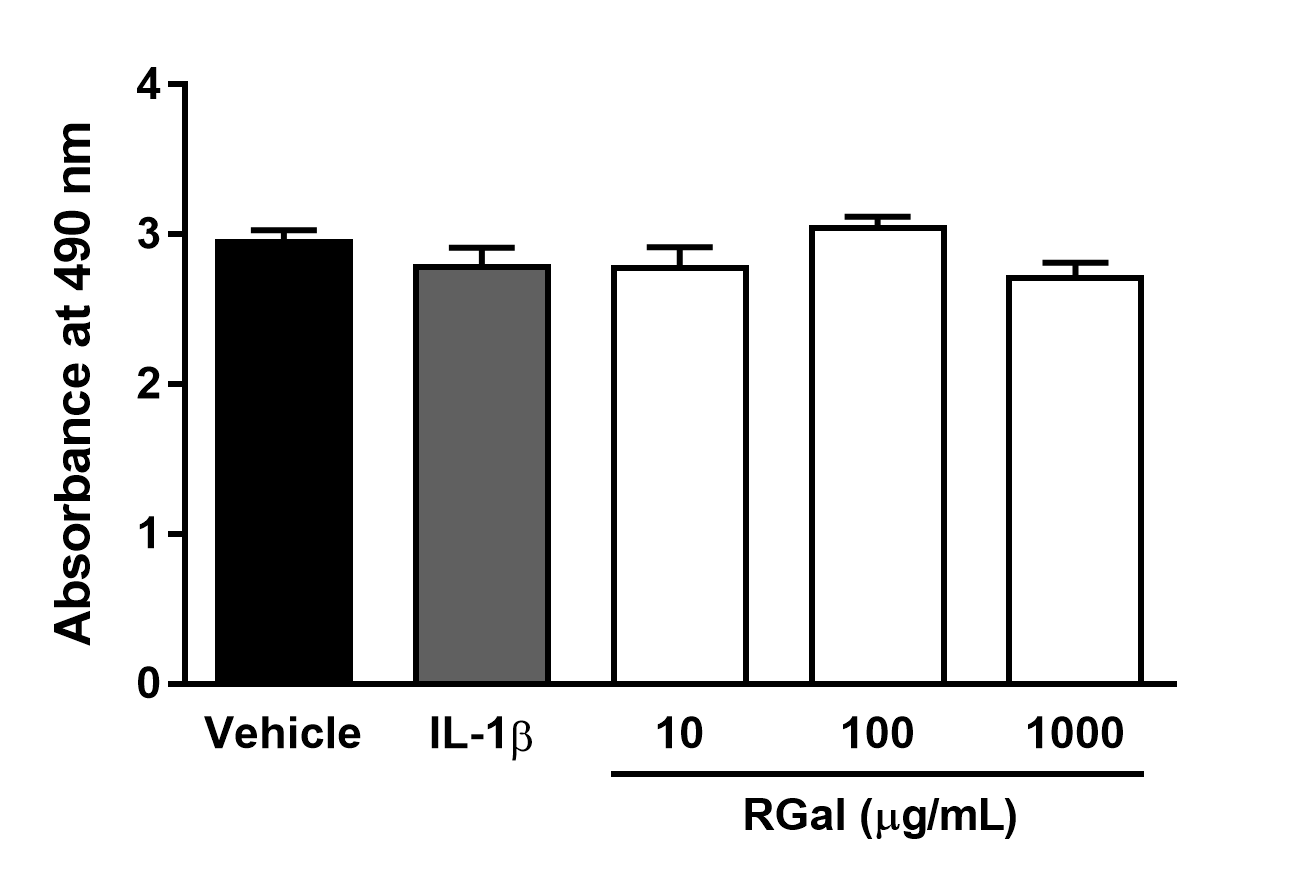
**
